# Finerenone in type 2 diabetes and renal outcomes: A random-effects model meta-analysis

**DOI:** 10.3389/fendo.2023.1114894

**Published:** 2023-01-20

**Authors:** Samit Ghosal, Binayak Sinha

**Affiliations:** ^1^ Nightingale Hospital, Kolkata, India; ^2^ Advanced Medical Research Institute (AMRI) Hospital, Kolkata, India

**Keywords:** PROSPERO 2022 CRD42022360003, type 2 diabetes, finerenone, meta-analysis, renal outcomes, type 2 diabetes

## Abstract

**Background:**

The nonsteroidal mineralocorticoid antagonist finerenone is a new addition to the list of agents (angiotensin converting enzyme inhibitors and sodium glucose cotransporter 2 inhibitors) conferring renal protection to patients with diabetic kidney disease. Two recent meta-analyses using the fixed effect model in patients with chronic kidney disease (both diabetic and nondiabetic populations) came to a conflicting conclusion on the effect of finerenone on eGFR decline. This meta-analysis was undertaken exclusively in the type 2 diabetes (T2D) population to explore the robustness and heterogeneity of the effect size by conducting a random effects model meta-analysis along with draft plots and prediction intervals.

**Materials and methods:**

A database search was conducted using the Cochrane library, PubMed, and Embase to identify relevant citations. Analysis was conducted on the 14^th^ of September 2022, using RevMan 5.4.1 and RStudio (2022.07.1, Build 554). The hazard ratio was used as the effect size for the renal composite, while the standardized mean difference (SMD) was used to estimate the effect size of eGFR decline and reduction in the urine albumin creatinine ratio (UACR). The Cochrane risk-of-bias was used to assess the quality of the studies. The primary outcome assessed was the renal composite defined as kidney failure, a sustained decrease of at least 40% in the eGFR from baseline, or death from renal causes.

**Results:**

A pooled population of 13,943 patients from four citations was included for analysis. The Cochrane risk of bias was used to assess the quality of the studies. There was a significant 16% reduction in the renal composite (kidney failure, a sustained decrease of at least 40% in the eGFR from baseline, or death from renal causes) [HR: 0.84, 95% CI 0.77-0.92, ^2^: 0, I^2^: 0%). Finerenone was also associated with reduction in UACR (SMD: -0.49, 95% CI -0.53 to -0.46, τ^2^: < 0.0001, I^2^: 0%, prediction interval: -0.57 to -0.41) and prevention of decline in eGFR (SMD: -0.32, 95% CI -0.37 to -0.27, τ^2^: < 0.0001, I^2^: 0%, prediction interval: -0.43 to -0.21) without any evidence for significant heterogeneity. Except for an increase in hyperkalaemia (RR: 2.22, 95% CI 1.93-2.24), adverse events were observed with fineronone compared to placebo (RR: 1.00, 95% CI 0.98-1.01).

**Conclusion:**

There are significant benefits in renal outcomes associated with finerenone treatment in T2D patients with established chronic kidney disease with a side effect profile comparable to placebo.

## 1 Introduction

Diabetes mellitus has emerged as the leading cause of chronic kidney disease (CKD), accounting for almost 60% of cases of CKD worldwide. Nearly 30%-40% of patients with type 2 diabetes (T2D) develop CKD (1). Despite the effective implementation of reductions in the individual risk factors associated with diabetic CKD, the overall mortality and progression to end-stage renal disease (ESRD) are increasing (1). The major contributors to mortality from CKD are atherosclerotic cardiovascular disease and heart failure (2).

Metabolic control lies at the heart of preventing the development of CKD. A reduction in HbA1c by 0.9% from baseline accounted for a 24%-33% reduction in the development of diabetic nephropathy (3). In addition to metabolic control, researchers have focused on specific targets and interventions to alter the development and progression of CKD.

There are several mechanistic pathways responsible for the inflammatory and fibrotic insult to the renal architecture and vasculature (4). Traditionally, it was postulated that the activation of the renin-angiotensin system (RAS) by triggers such as insulin resistance resulted in ligand-dependent activation of the mineralocorticoid (MR) receptor by aldosterone, leading to salt retention and hypertension and consequent renal injury. This led to the strategy of RAS blockage, which was met with some success in reducing cardio-renal outcomes (5). However, with the discovery of “escape pathways” (incomplete RAS inactivation by RAS blockade), attempts were made to use dual blockade (ACEi and ARB) as well as direct renin blockade to obtain better cardio-renal benefits. Both these attempts proved to be abortive (6, 7). The recent understanding of nonligand-based activation of MR by Ras-related C3 botulinum toxin substrate 1 (Rac 1), hyperglycaemia, and excess sodium brought strategies blocking MR into focus (8). It seems plausible that additional nephropretection can be achieved with the concomitant use of MR antagonists in addition to ACEi/ARBs.

Nonsteroidal MR antagonists (MRA) were chosen since, being more MR selective, they are less likely to cause hyperkalaemia in contrast to steroidal MRA. This is obviously more suitable in the management of CKD (9). The dose finding ARTS (MinerAlocorticoid Receptor antagonist Tolerability Study) documented a significant impact of finerenone on albuminuria progression at doses of 10 mg-20 mg per day (10). With the adequate dose identified and its positive result on the cardio-renal components documented, finerenone was given FDA approval in 2021 for use in T2D patients with an aim to reduce cardio-renal adverse outcomes (11).

There were a couple of meta-analyses estimating the effectiveness of finerenone versus placebo on the renal composite as well as the individual components of urine albumin creatinine ratio (UACR) and estimated glomerular filtration rates (eGFR) (12, 13). Although both meta-analyses documented similar benefits regarding renal composite, there was discordance as far as benefits on eGFR reduction were concerned. Moreover, of the five included citations, one by Pitt el al did not include patients with T2D, while the other four did.

To explore the impact of finerenone on renal outcomes exclusively in the T2D population, this meta-analysis was undertaken. We intended to examine the data limited to the T2D population and look for any heterogeneity in the outcomes that could explain the divergent results in the two meta-analyses.

## 2 Materials and methods

This meta-analysis was conducted according to the recommendations of the PRISMA statement and registered with PROSPERO (CRD42022360003) (14).

### 2.1 Literature searches, search strategies and eligibility criteria

The web search was conducted using the PICO search pattern:

P: Type 2 diabetic patients with established chronic kidney disease

I: Finerenone

C: Control

O: (a). Renal composite (kidney failure, a sustained decrease of at least 40% in the eGFR from baseline, or death from renal causes), (b). Decrease in urine albumin creatinine ratio (UACR), (c). eGFR decline, (c). Adverse events associated with the active intervention (any adverse event, hyperkalemia, and acute renal injury).

### 2.2 Data extraction included assessment of the quality of the studies

Both authors conducted a web-based search for relevant citations dependent on the selected keywords. Additional filters included a cap on age above 18 years and clinical trials. No restrictions were placed based on language or date of publication. SG conducted the meta-analysis.

Having identified the four citations to be taken up for analysis, the data required for both analyses were entered into an Excel sheet. The chances of any error in entering the data were cross-checked by another author (BS). The quality of the selected citations was assessed using the Cochrane risk-of-bias algorithm, which included random sequence generation, allocation concealment, blinding of participants and personnel, blinding of outcome data, incomplete outcome data, selective reporting, and other biases. All the selected citations were evaluated along with their supplementary data and scored individually by BS & SG. Any dispute was reassessed by SG and BS, and a final decision was made by consensus. Individual publication bias was analyzed using funnel plots. (**Supplementary Figure S2**)

After the initial process, a manual search was conducted jointly to identify the citations that met the inclusion criteria:

- Randomized controlled trials.
- Age limit: above 18 years, with type 2 diabetes mellitus and documented CKD defined as a UACR more than 200 mg/g or a decline in eGFR (<60 ml/min) for at least three months.
- Inclusion of placebo as the control arm.
- A minimum of 12 weeks of follow-up.
- Reporting of prespecified renal events (renal composite, UACR and eGFR).
- Reporting of adverse events related to the active intervention.

Exclusion criteria:

- Non-randomised trials, abstracts, review articles, and case reports were excluded from analysis.
- Age of recruitment below 18 years.
- A follow-up duration less than 3 months.
- Inclusion of active intervention in the comparator arm capable of influencing the renal outcomes.
- Any other type of diabetes (Type 1 diabetes, post-pancreatitis secondary diabetes).
- A clear documentation of exclusion of all acute renal events.

### 2.3 Patient approval and clearance from the ethical committee

In this systematic review and meta-analysis, there was no direct handling of patients. In addition, effect size estimates that were already published and in open web-based domains were used to conduct the meta-analysis. As a result, there was no requirement for patient or ethical committee consent.

### 2.4 Statistical analysis

The hazard ratio (HR) and standardized mean difference (SMD) risk were used as the preferred parameters of interest because of the differing patterns in reporting outcomes of interest in the included citations. The renal composite effect size was reported in two studies in the form of HR, and hence, the pooled mean effect size was analysed using HR. However, the other outcomes of interest (UACR and eGFR) were reported as raw mean values, and hence, an SMD was used to estimate the pooled effect size. Risk ratio was used as the effect size estimate for assessment of the adverse events since raw events were reported. In addition to the effect size, hypothesis testing was performed and reported in the form of a 95% confidence interval (95% CI) and p value. Since the p value does not represent the effect size distribution spectrum, a prediction interval was also planned to be assessed along with representation of the p value function distribution in the form of a drapery plot. The summary of results was reported as a forest plot, which also included the weightage of the individual studies. The analysis was conducted using the RevMan 5.4.1 and R studio (2022.07.1, Build 554) platforms. Heterogeneity was assessed using the prediction interval, Q statistic and Higgin’s I^2^ test. Heterogeneity was defined as a Q statistic value exceeding the degrees of freedom with a significance cut off p=0.1.

In view of the diverse population represented in the citations as well as selection of the citations from a universe of available data, a random effects model was used to estimate the effect size. This strategy also facilitated the process of assessing heterogeneity in contrast to the fixed effect model. A sensitivity and subgroup analysis were planned if significant heterogeneity related to the pooled effect size was encountered.

Prominent adverse events associated with finerenone compared to placebo were analysed using the risk ratio as the effect size. Since, UACR and assessment of eGFR are routinely performed in clinical practice, we included them as part of subgroup analysis. Both UACR as well as eGFR decline were part of the renal composite assessed as the primary outcome of interest.

## 3 Results

### 3.1 Identification of studies for analysis

**Figure 1:**
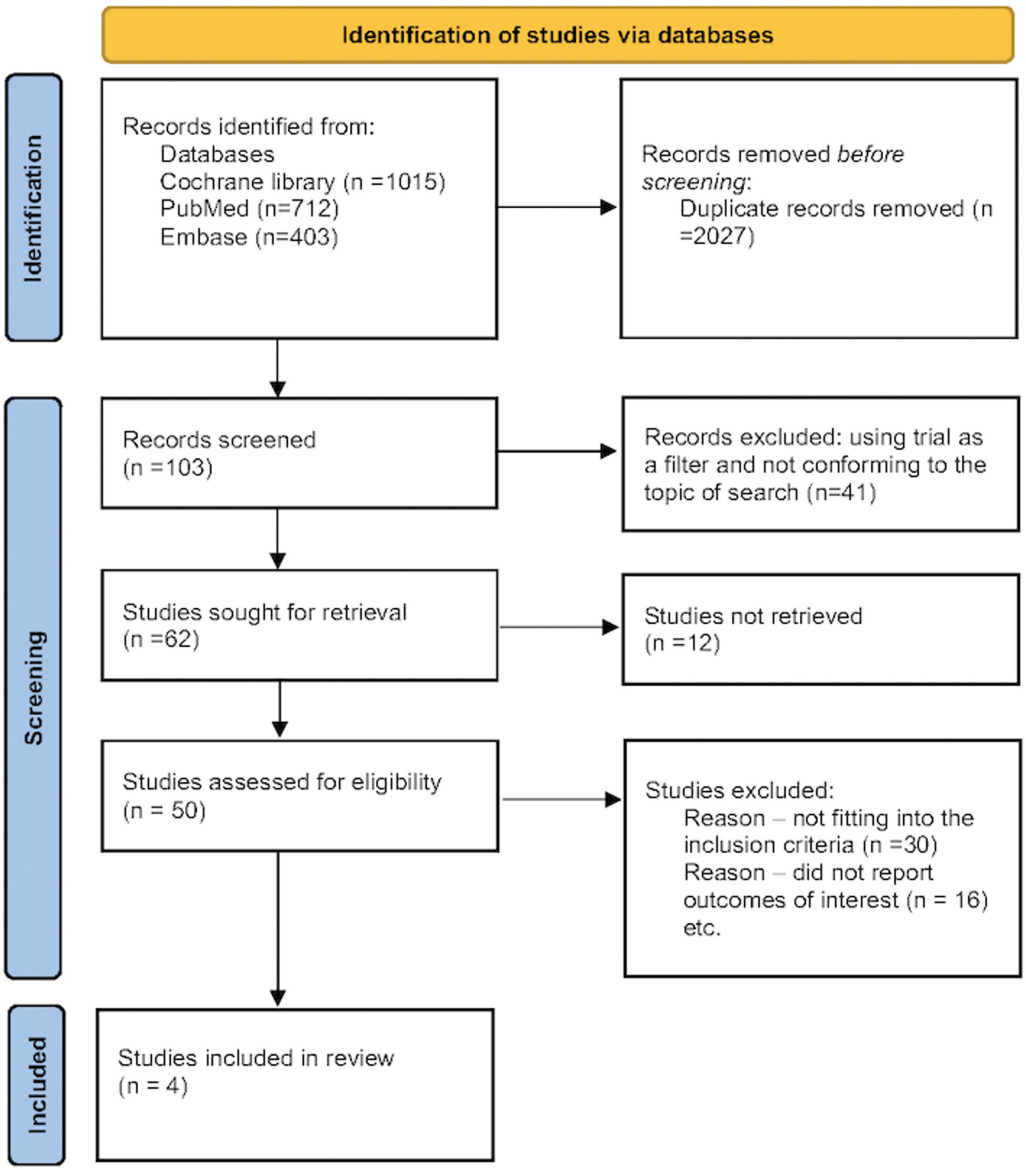
The study selection process.

The randomized prospective studies were identified through a thorough database search (Cochrane Library, PubMed, and Embase). The search was divided into three categories: (a) related to the intervention in question “Finerenone”, “Nonsteroidal mineralocorticoid antagonist”, (b) related to the primary disease in question “Type 2 Diabetes mellitus”, “T2DM”, “Chronic kidney disease”, “CKD”, “Diabetic kidney disease”, “DKD”, and (c) related to the outcomes assessed, which included the terms “Diabetic kidney disease”, “Urine albumin creatinine ratio”, “UACR”, “eGFR decline”, and “Renal composite”). Furthermore, the primary search filters included human data and clinical trials, although no search restrictions on time or language were used. While performing the Cochrane library search, the outcome keywords [(a), (b), and (c)] were clubbed using Boolean OR. The search results were then combined using Boolean AND to yield the first set of citations. The initial search was followed up by a detailed manual search. The main purpose of the manual search was to screen the initially filtered citations for duplicate publications, remove review articles, as well as identify those citations not conforming to the predetermined inclusion and exclusion criteria (**Figure 1**). The full search strategy is available in **Supplementary Appendix 1**.

Any citation that compared finerenone versus placebo was included for analysis.

### 3.2 Baseline characteristics of the studies

The preliminary web search resulted in the identification of 2130 citations, among which many duplicate (n=2027) studies were identified and removed. The screening process and search for relevant citations using the inclusion criteria resulted in the selection of four citations for the final analysis (15–18). A study by Pitt et al. (2013) was excluded because it was not conducted on patients with diagnosed T2D (10). The study by Bakris et al. (2015) was conducted on T2D patients with significant albuminuria optimized on an angiotensin-converting enzyme inhibitor or an angiotensin receptor blocker and primarily evaluated for reduction in UACR (15). The FIDELIO-DKD trial (Bakris et al., 2020) was conducted on patients with T2D with established DKD and primarily evaluated the impact of finerenone on the renal composite (16). The study by Katayama et al. evaluated the impact of finerenone on reduction of UACR at 90 days on T2D patients with diabetic nephropathy (17). The FIRAGO-DKD study (Pitt et al., 2021) expanded the scope of the inclusion criteria of the FIDELIO-DKD study by recruiting T2D patients with moderate albuminuria and stage 2-4 CKD (18). The primary aim of this study was to evaluate the impact of finerenone on the renal composite (kidney failure, a sustained decrease of at least 40% in the eGFR from baseline, or death from renal causes) as well as reduction in UACR and retardation of decline in eGFR. The meta-analysis was conducted on a pooled patient population of 13,943 from 4 citations, divided into 7330 individuals on finerenone and 6613 patients on placebo. The duration of follow-up ranged from 3 to 40.8 months. The baseline characteristics of the citations included in the analysis are summarized in **Table 1**. Patients receiving a dose of finerenone ≥ 10 mg/day were included in the analysis.

**Table 1:** Baseline characteristics of the citations included for analysis.

Study	Year	Male/Female (Finerenone arm)	Mean Age (years)	Finerenone (n)	Finerenone ≥10 mg/day (n)	Baseline UACR/eGFR *	Placebo (n)	Follow up (months)
Bakris et al. (15)	2015	570/157	64.7 ± 9.26	727	342	202.7/66.0	94	3
Bakris et al. (16)	2020	1953/880	65.6 ± 9.1	2833	2833	302/67.6 ± 21.7	2841	31.2 (median)
Katayama et al. (17)	2017	67/17	64.0 ± 8.26	84	36	833/44.4 ± 12.5	12	3
Pitt et al. (18)	2021	2528/1158	64.1 ± 9.8	3686	3686	127.67/61.48	3666	40.8 (median)

### 3.3 Outcome measures: Renal composite

**Figure 2:**
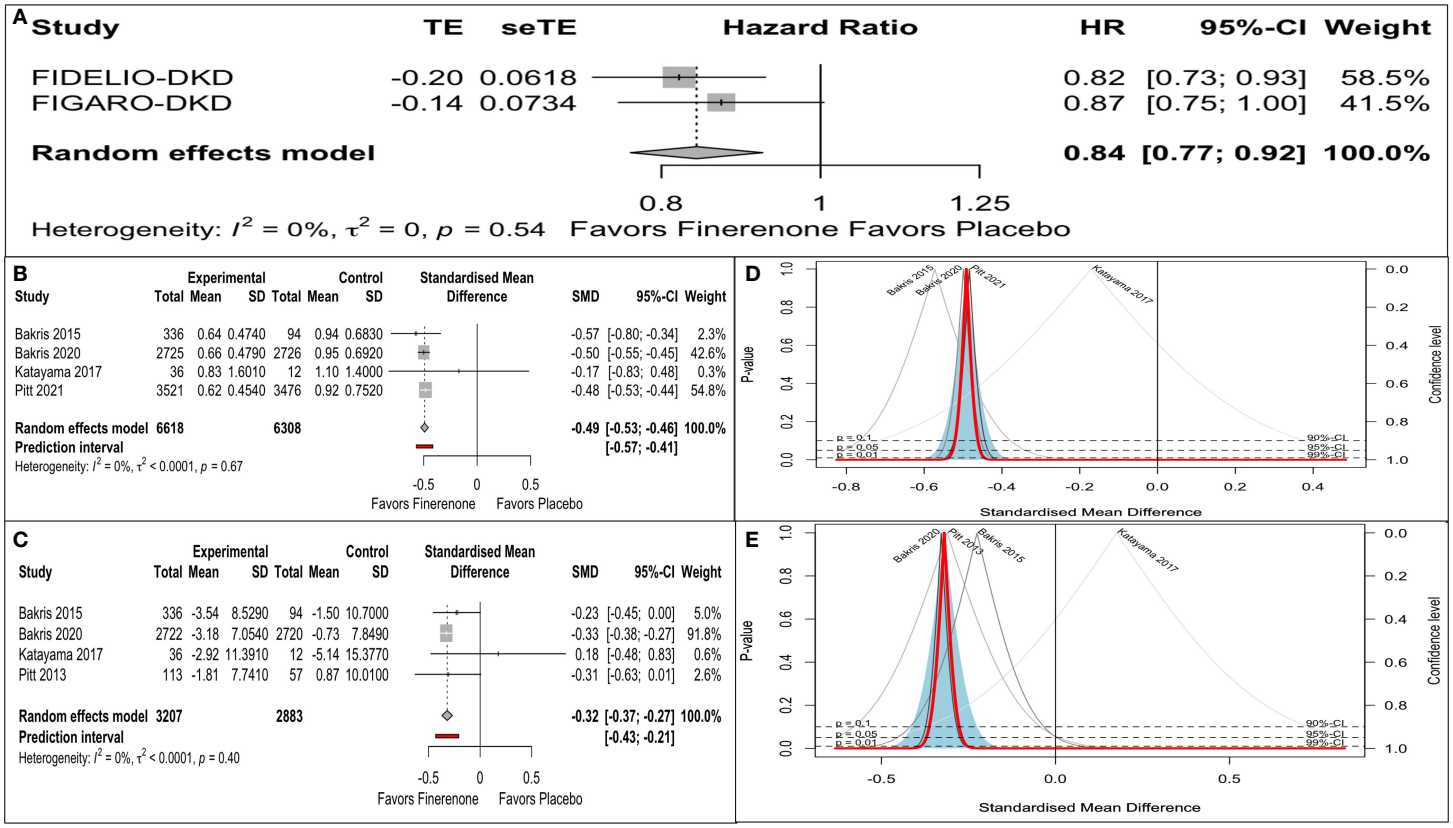
Finerenone versus placebo on: **(A)** Renal composite (Forest plot): hazard ratio (HR). **(B)** Reduction in UACR (Forest plot): Standardized mean difference (SMD). **(C)** eGFR decline (Forest plot): Standardized mean difference (SMD). **(D)** eGFR decline (Drapery plot). **(E)** Reduction in UACR (Drapery plot).

Only two of the four citations reported renal composite conforming to a universal definition. The pooled estimate of the mean effect size was significant, as indicated by a 16% reduction in the renal composite (HR: 0.86, 95% CI 0.77-0.92). A Q statistic of 0.38 with a df of 1 indicated the absence of significant heterogeneity in the true effect size (**Figure 2**). The prediction interval could not be assessed for the renal composite in view of the requirement of at least three studies.

### 3.4 Outcome measures: UACR

There was an impressive reduction in UACR from baseline in the finerenone arm compared to placebo (SMD: -0.49, 95% CI -0.53 to -0.43). A predictive interval of the effect size (-0.57 to -0.41) ruled out significant uncertainty in the true effect size (**Figure 2**).

### 3.5 Outcome measures: eGFR decline

There was an impressive retardation in eGFR decline from baseline in the finerenone arm compared to placebo (SMD: -032, 95% CI -0.37 to -0.27). A predictive interval of the effect size (-0.43 to -0.21) complemented the significant impact on the mean effect size (**Figure 2**).

### 3.6 Adverse events

**Figure 3:**
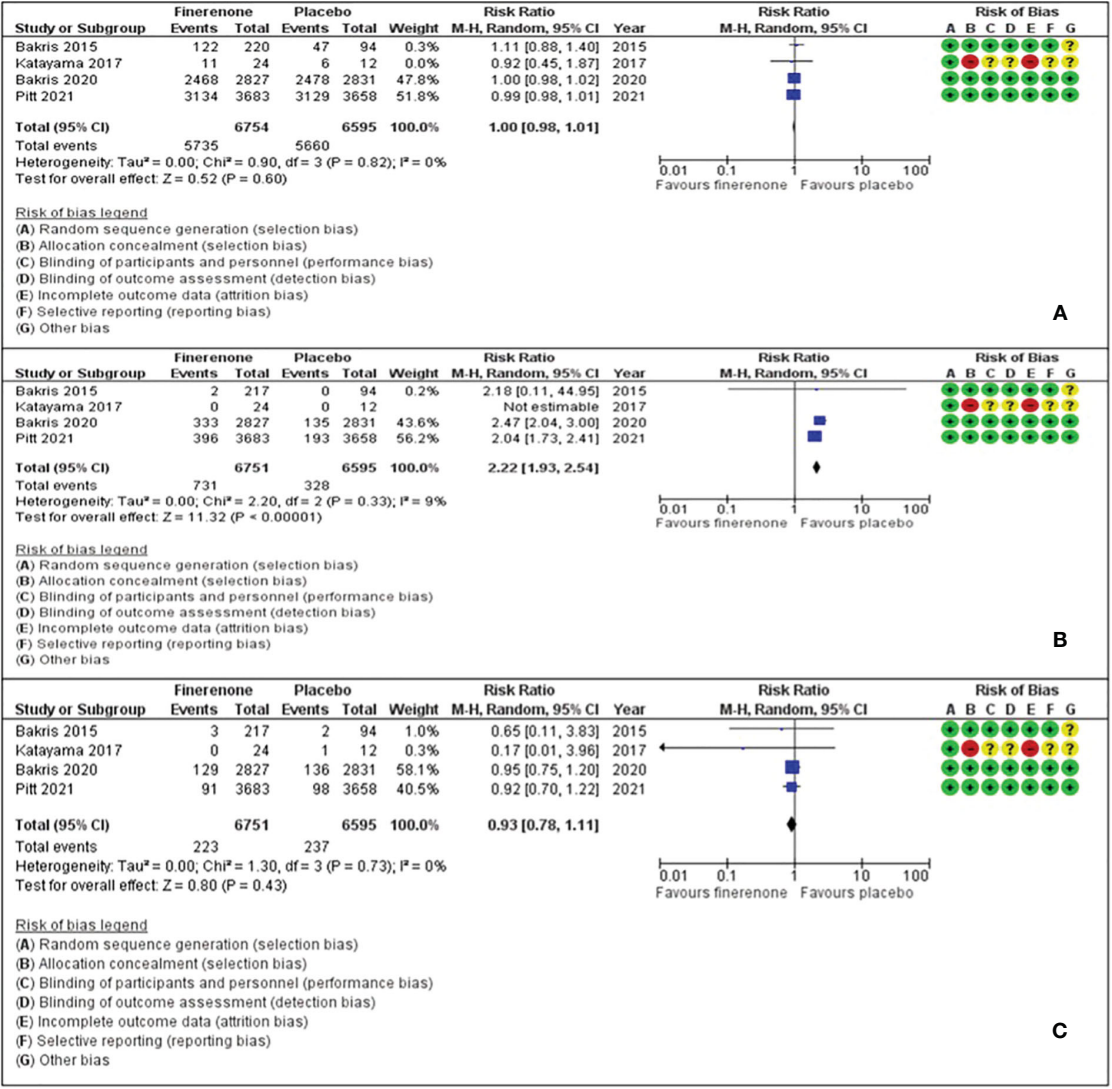
Effect of finerenone versus placebo on: **(A)** Any adverse events. **(B)** Hyperkalaemia. **(C)** Acute renal injury or eGFR decline of ≥40%.

Adverse events with finerenone were comparable to placebo (RR: 1.00, 95% CI 0.98-1.01), as was the risk of acute kidney injury or an eGFR decline of ≥40% (RR: 0.98, 95% CI 0.78-1.11). However, the risk ratio of hyperkalaemia was significantly higher with finerenone (RR: 2.22, 95% CI 1.93-2.24) (**Figure 3**).

## 4 Discussion

Diabetic nephropathy is one of the leading causes of mortality and morbidity in patients with T2D. Targeting albuminuria and retarding the decline in eGFR have become the therapeutic strategy of choice in preventing cardiorenal outcomes. RAS inhibition remained the backbone of such a strategy. However, less than 50% of the target population in the RAS blockade arm of clinical trials achieved their primary endpoint in most of these trials, creating scope for newer strategies to tackle this unmet need (5). Recently, SGLT-2is and GLP1-RAs have been found to have a positive impact on cardio-renal outcomes (19). However, the search for taming the deleterious effects of aldosterone on target organs through activation of MR was not abandoned. Aldosterone is elevated approximately 4-fold in patients with CKD-Stage 4 or less, leading to inflammation and fibrosis in the tissues of target organs (20). Because MR is expressed in both the kidneys and the heart, MR receptor antagonism is considered as a prominent strategy to alter adverse cardio-renal outcomes (21). Nonsteroidal MRA are more selective in nature and less prone to gynaecomastia and hyperkalaemia. Finerenone is a prototype of an MR antagonist.

### 4.1 Literature review

The phase II program ARTS (MinerAlocorticoid Receptor antagonist Tolerability Study) was conducted in CKD patients with chronic heart failure and reduced ejection fraction (HFrEF), with spirololactone as a comparator (12). There was a comparable reduction in pro-brain natriuretic peptide (NT-Pro BNP) as well as UACR, with less gynaecomastia and hyperkalaemia. The ARTS-DN (MinerAlocorticoid Receptor antagonist Tolerability Study in Diabetic Nephropathy) comparing finerenone versus placebo documented a dose-dependent reduction in albuminuria at 90 days without a significant impact on eGFR decline (15). The FIDELIO-CDK study documented impressive benefits with the use of finerenone in reducing composite renal outcomes (kidney failure, a sustained decrease of at least 40% in the eGFR from baseline, or death from renal causes) in patients with T2D and established CKD (16). The FIRAGO-DKD study was the first to show a positive cardiovascular result using a 4-point MACE (composite of death from cardiovascular causes, nonfatal myocardial infarction, nonfatal stroke, or hospitalization for heart failure) in T2D patients with established CKD (18). A meta-analysis of five RCTs by Zhang et al. using the fixed effects model demonstrated a significant improvement in UACR reduction as well as retardation of eGFR decline with finerenone compared to placebo (12). However, another meta-analysis by Fu et al. with four studies using the fixed effects model demonstrated a positive impact of finerenone on UACR reduction but not on the retardation of eGFR decline (13).

### 4.2 Findings from our study

Our meta-analysis was conducted exclusively on T2D patients with established CKD. This is probably the first meta-analysis to explore the impact of finerenone on renal outcomes in T2D patients. In addition, we used the random effect model to conduct the meta-analysis, especially since there were significant differences in the baseline characteristics of the patients recruited in the individual studies, and to assess any heterogeneity and uncertainty in the true effect size. We documented a significant 16% reduction in the renal composite (kidney failure, a sustained decrease of at least 40% in the eGFR from baseline, or death from renal causes) [HR: 0.84, 95% CI 0.77-0.92, τ^2^: 0, I^2^: 0%) in the T2D patients with CKD receiving finerenone compared to the placebo arm. There was also an impressive reduction in UACR (SMD: -0.49, 95% CI -0.53 to -0.46, τ^2^: < 0.0001, I^2^: 0%, prediction interval: -0.57 to -0.41) and retardation of decline in eGFR (SMD: -0.32, 95% CI -0.37 to -0.27, τ^2^: < 0.0001, I^2^: 0%, prediction interval: -0.43 to -0.21) in the finerenone arm without any evidence for significant heterogeneity. Our meta-analysis endorses the robustness of the renal benefits in the T2D cohort as suggested in the RCTs, without any heterogeneity or uncertainty in the true effect size as assessed with the prediction interval. In addition, this study illustrated that fineronone usage was not associated with any adverse outcome or worsening of renal disease or acute kidney injury. However, as expected, since fineronone is after all an MRA, there is a significant increase in the rates of hyperkalaemia.

### 4.3 Limitations and strengths

Our meta-analysis has a few limitations. First, we analysed the reported mean data and did not have access to individual patient data. As a result, the bias ascertainment could have been impacted, since we were assessing data from published materials. Second, there are a couple of studies of very short duration in which the dramatic nature of the impact on outcomes could have skewed the overall result. Third, although renal composite was reported in a couple of trials on a time-dependent basis (HR), the reporting of UACR and eGFR decline were not time-dependent in nature.

The main strength of this meta-analysis was that it was conducted using the random effects model, taking into consideration the heterogeneity associated with the type of included citations and baseline characteristics of the patients. Another advantage was the use of the prediction interval, which helped us to assess any uncertainty in the true effect size. Finally, we segregated the T2D patients with CKD, unlike the pooled CKD analysis performed in previous meta-analyses, resulting in more specific reporting of the results.

## 5 Conclusion

The nonsteroidal MRA finerenone, by way of its unique mechanism of action, is a novel strategy in the fight against the progression of diabetic CKD. This meta-analysis convincingly demonstrates that finerenone not only produces an impressive reduction in the renal composite but also reduces UACR and retards the decline in eGFR in patients with T2D who have established CKD. Reassuringly, fineronone is well tolerated, except for an increased risk of hyperkalaemia, which must be monitored during fineronone therapy.

## Ethics approval and consent to participate

We analyzed published data; hence, ethical approval was not needed. In addition, we did not deal with patients, and hence consent to participate was not applicable.

## Data availability statement

The original contributions presented in the study are included in the article/**Supplementary Material**. Further inquiries can be directed to the corresponding author.

## Author contributions

SG and BS conceptualized the study. The meta-analysis was conducted by SG. BS cross validated the data and prepared the manuscript. SG and BS conducted an expanded literature review to contextualize the results from the meta-analysis. All authors contributed to the article and approved the submitted version.

## Supplementary material

Supplementary Figure 1 Risk of bias assessment using the Cochrane risk-of-bias algorithm.
