# Supplementary material for: Finerenone in type 2 diabetes and renal outcomes: A random-effects model meta-analysis
Source: Front Endocrinol (Lausanne). 2023 Jan 20;14:1114894. doi: 10.3389/fendo.2023.1114894 (PMC9895809; doi:10.3389/fendo.2023.1114894)
Supplement: Supplementary file 1 [file Table_1.docx]

Supplementary Appendix 1:

Search Name: Kerendia

Last Saved: 14/09/2022 09:16:11

Comment: Meta analysis

ID Search

#1 (Finerenone):ti,ab,kw (Word variations have been searched)

#2 (Non-steroidal mineralocorticoid antagonist):ti,ab,kw (Word variations have been searched)

#3 (Urine albumin creatinine ratio):ti,ab,kw (Word variations have been searched)

#4 (eGFR):ti,ab,kw (Word variations have been searched)

#5 (Chronic kidney disease):ti,ab,kw (Word variations have been searched)

#6 (Diabetic kidney disease):ti,ab,kw (Word variations have been searched)

#7 (DKD):ti,ab,kw (Word variations have been searched)

#8 (CKD):ti,ab,kw (Word variations have been searched)

#9 (RENAL COMPOSITE):ti,ab,kw (Word variations have been searched)

#10 (Type 2 diabetes mellitus):ti,ab,kw (Word variations have been searched)

#11 (T2DM):ti,ab,kw (Word variations have been searched)

#12 #1 OR #2

#13 #5 OR #6 OR #7 OR #8

#14 #10 OR #11 in Trials

#15 #13 AND #14

#16 #3 OR #4 OR #9

#17 #15 AND #16 in Trials

#18 #15 AND #16 in Trials
