# Supplementary figures and images for: Finerenone in type 2 diabetes and renal outcomes: A random-effects model meta-analysis
Source: Front Endocrinol (Lausanne). 2023 Jan 20;14:1114894. doi: 10.3389/fendo.2023.1114894 (PMC9895809; doi:10.3389/fendo.2023.1114894)

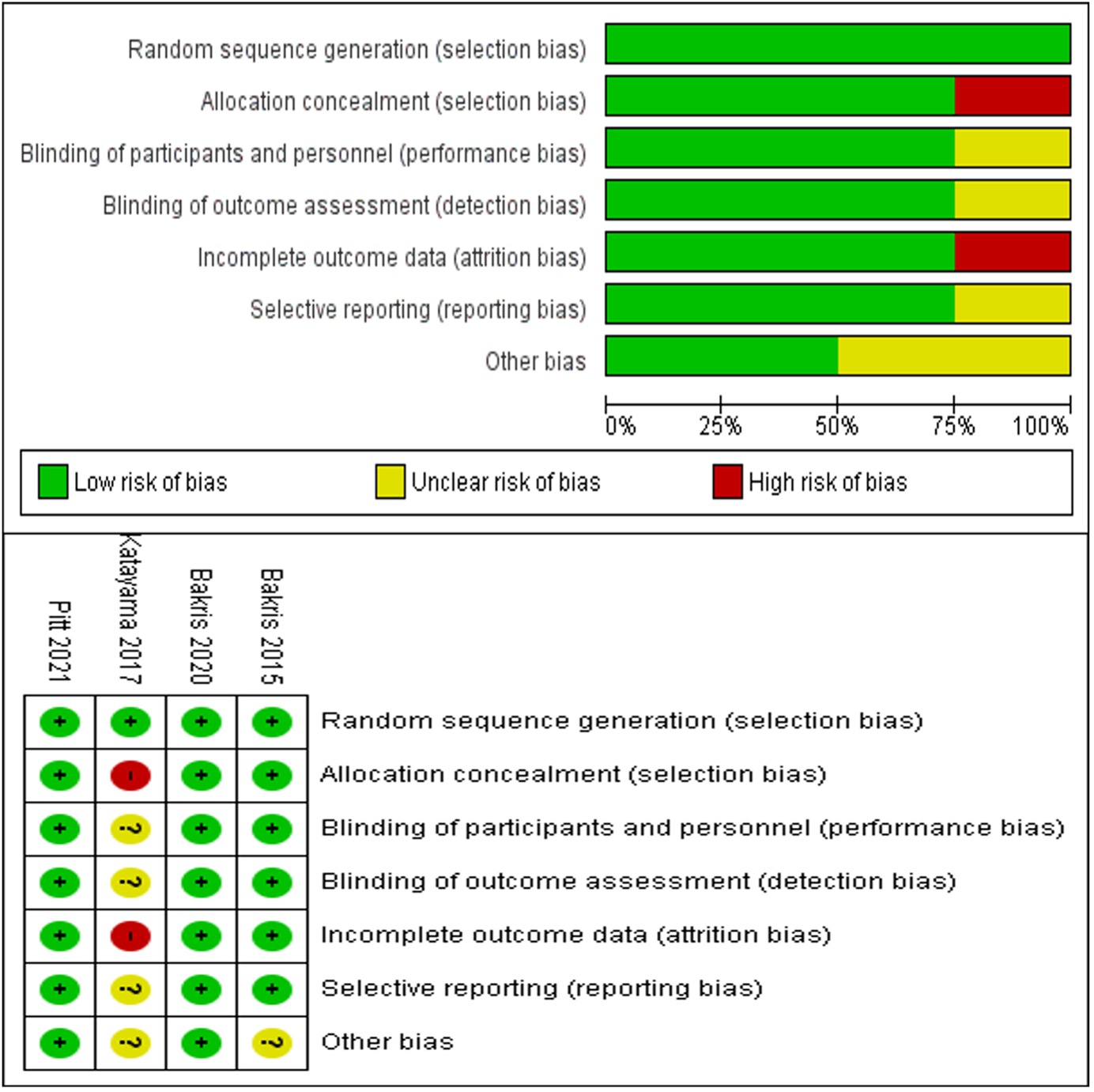

Supplement: Supplementary Figure 1 — Risk of bias assessment using the Cochrane risk-of-bias algorithm. [file Image_1.jpeg]
